# Integrated metabolomic and transcriptomic analysis of the mechanism underlying leaf variegation in *Miscanthus sinensis* ‘Zebrinus’

**DOI:** 10.3389/fpls.2026.1748715

**Published:** 2026-02-11

**Authors:** Yu-Lan Han, Yi-Shan Cheng, Yi-Xin Li, Deng-Jin Luo, Ming Cai, Lan Mu

**Affiliations:** 1 College of Landscape Architecture and Horticulture Sciences, Southwest Forestry University, Kunming, China; 2 Yunnan International Joint Research and Development Center for Integrated Utilization of Ornamental Grass, Kunming, China; 3 Yunnan Academy of Grassland and Animal Science, Kunming, China

**Keywords:** leaf variegation, *Miscanthus sinensis ‘Zebrinus’*, metabolomics, transcriptomics, differential metabolites, differentially expressed genes

## Abstract

**Introduction:**

*Miscanthus sinensis* ‘Zebrinus’ is a landscape plant with high ornamental value, whose core ornamental feature is determined by the irregularly distributed yellow variegation on its leaves, supporting its extensive application in landscape design and configuration. *M. sinensis* ‘Zebrinus’, as a typical variegated-leaf gramineous plant, possesses a key phenotypic trait of leaf variegation that distinguishes it from ordinary *Miscanthus* species. However, up to the present moment, we know little about the molecular regulatory mechanism underlying this unique variegation, with relevant research carried out in the exploratory stage.

**Methods:**

This study was performed with the use of two leaf phenotypes [Yellow area of variegated leaves (YS) and Green area of variegated leaves (GS)] of *M. sinensis* ‘Zebrinus’. Differential metabolites between GS and YS leaf samples was conducted using the metabolomic analysis, with a focus on identifying key metabolites associated with leaf variegation. Furthermore, gene expression profiles of GS and YS leaves were acquired through transcriptome sequencing. With the screening of differentially expressed genes (DEGs), this study also carried out functional annotation and pathway enrichment analysis. Moreover, the expression levels of candidate genes in GS and YS leaves were measured via quantitative real-time polymerase chain reaction (qRT-PCR). In addition, a “gene-metabolite” regulatory network was constructed by integrating the metabolomic and transcriptomic data to screen out the key metabolites and core genes responsible for regulating leaf variegation in *M. sinensis* ‘Zebrinus’.

**Results:**

Metabolomic analysis identified 4,036 common metabolites in GS and YS samples, with major enrichment in the flavonoid biosynthesis pathway. Secondary classification of this pathway indicated that flavonoids had the highest content. Further comparison of the expression levels of key metabolites revealed that the accumulation patterns of neohesperidin, taxifolin, naringenin, and xanthohumol in YS were all higher than those in GS, with naringenin showing the most significant difference, suggesting that it might be the core metabolite regulating leaf spot formation. According to subsequent transcriptome sequencing, 5,252 DEGs were screened out from the YS and GS samples, which were mainly enriched in flavonoid biosynthesis phenylpropanoid biosynthesis and other pathways. qRT-PCR presented the highest expression level in chalcone synthase (*CHS*). Integration of metabolome and transcriptome demonstrated significant enrichment of differential metabolites and DEGs in the flavonoid biosynthesis pathway. Additionally, correlation network graph analysis suggested the highest correlation of naringenin with *CHS*.

**Discussion:**

This study identifies the core intrinsic regulatory mechanism underlying leaf variegation in *M. sinensis* ‘Zebrinus’ through integrated metabolomic and transcriptomic analysis. *CHS* has a strong correlation with naringenin, suggesting that the transcriptional regulation of the *CHS* gene may directly affect the biosynthesis of naringenin. The synergistic effect of the two may be one of the key molecular mechanisms underlying the formation of yellow leaf variegation.

## 1 Introduction

Leaf variegation is one of the important phenotypes in ornamental plants, which helps plants avoid pest infestation (Lev-Yadun, 2021), and endows them with high ornamental value (Ran et al., 2024). *M. sinensis* ‘Zebrinus’ is a herbaceous plant with both ornamental value and ecological application potential, which is distinguished from ordinary *Miscanthus* species by its unique leaf variegation trait. Meanwhile, in other plants, common leaf variegation shapes include V-shaped (*Trifolium repens*), fishbone-shaped (*Primulina pungentisepala*), feather-shaped (*Ficus benjamina* ‘Variegata’), and punctated (*Codiaeum variegatum*), etc. Compared with the aforementioned diverse leaf variegation shapes, the yellow stripes on the leaf variegation of *M. sinensis* ‘Zebrinus’ are unique. Beyond the ornamental appeal of landscape configurations, this trait also provides an ideal material for studying the mechanisms underlying plant leaf variegation. As has been documented, leaf variegation is the result of the synergistic interaction between plant gene expression regulation and metabolic pathways, involving multiple biological processes such as pigment synthesis (Liu et al., 2021), cell structure development (Yang et al., 2015), and environmental response (Zhao et al., 2020; Li et al., 2019; Chen et al., 2022). Therefore, it is of great significance to elucidate its formation mechanism for revealing the molecular basis of plant phenotypic variation.

To date, the formation of leaf variegation in variegated-leaf plants is generally studied on a single level. For instance, variegation-related differentially expressed genes (DEGs) have been identified previously through transcriptomic analysis, involving pathways such as photosynthetic systems and pigment metabolism, yet with the lack of direct validation at the metabolite level (Yang et al., 2015; Zhao et al., 2020). Meanwhile, some other researchers explored metabolite differences between variegated and normal leaf regions, yet without the establishment of a close link to the regulatory mechanisms of gene expression. It should be acknowledged that leaf variegation is a comprehensive manifestation of dynamic changes in metabolic networks under genetic regulation (Shen et al., 2018). Consequently, it may be a challenge to fully elucidate its complex mechanisms from the transcriptomic or metabolomic level solely.

With the advancement of metabolomics and transcriptomics in recent decades, researchers have acquire a new perspective for in-depth understanding of the relationship between plant metabolism and gene expression. Metabolomics can detect changes in the types and contents of metabolites in leaves (Zhao XC, et al., 2024), while transcriptomics enables the analysis of gene expression levels and identification of key genes involved in metabolic pathways that regulate leaf color (Wolf, 2013). An integration of these two methods can achieve a systematic elucidation of the molecular basis of leaf variegation at the “gene-metabolite” level, which has now been extensively applied in the research of leaf-color plants. For example, integrated metabolomic and transcriptomic analyses have been applied and identified significant upregulation of the dihydroflavonol 4-reductase (*DFR*) gene in yellow leaves of *Ginkgo biloba*. As a key regulatory node in the anthocyanin biosynthesis pathway, *DFR* can affect the synthesis and metabolic pathways of anthocyanins in a direct or indirect manner, leading to changes in epicatechin content in plants and thus the formation of mutant leaves in *G. biloba* (Wu et al., 2020; Li et al., 2012). Meanwhile, in *Osmanthus fragrans* ‘Ziyan Gongzhu’, the formation of fuchsia and light purple leaves might be related to the low expression of chlorophyll synthesis genes (e.g., *HEMA*, *CHLG*, and *CAO*) and high expression of anthocyanin synthesis genes (e.g., *F3H*, *F3’H*, *DFR*, and *ANS*), among which cyanidin might be the pigment closely related to leaf color (Guo et al., 2023). In *Rhododendron rex*, the upregulation of chalcone synthase (*CHS*), chalcone flavanones (*CHI*), flavonoid 3-hydroxylase (*F3H*), and *DFR* as well as downregulation of hydroxycinnamoyl-CoA shikimate/quinate hydroxy cinnamoyl transferase (*HCT*) and flavonol synthase (*FLS*) would promote the accumulation of anthocyanins (e.g., pelargonidin, cyanidin 3, and delphinidin) in petal spots (Wang SQ, et al., 2025). Currently however, there is a scarcity of systematic research on herbaceous plants using integrated metabolomic and transcriptomic analyses, despite extensive application in studying leaf color mutation in woody plants (Zhang et al., 2024; Wang et al., 2023), peel color analysis (Shen et al., 2019; Dang et al., 2023), flower variegation (Wang SQ, et al., 2025; Luan et al., 2024), etc. Therefore, it highlights the necessity for carrying out in-depth studies on the mechanism of leaf variegation in herbaceous plants, thereby improving the theory of plant color regulation.

Although the leaf stripe trait of *M. sinensis* ‘Zebrinus’ makes it an ornamental herbaceous plant with great application value, the molecular regulatory mechanism underlying stripe phenotype formation remains unclear. Currently, there is a lack of systematic analysis on the core metabolic basis of stripe formation in *M. sinensis* ‘Zebrinus’. Neither has a comprehensive comparison been made between the metabolite profiles of the yellow areas and the green areas of the leaves, nor have the key metabolites driving stripe phenotype formation and their accumulation patterns been identified. At the transcriptional regulation level, differentially expressed genes in pathways closely related to stripe formation, such as pigment synthesis, have not been systematically explored, and the regulatory role of key structural genes remains unknown. More critically, there is a significant gap in the omics correlation analysis between the metabolome and transcriptome, making it impossible to establish an intrinsic connection of “differential gene expression - key metabolite accumulation - stripe phenotype formation”, thus making it difficult to elucidate the core regulatory pathway of stripe formation in *M. sinensis* ‘Zebrinus’.

Based on the aforementioned research status, this study utilizes the striped and normal green regions of *M. sinensis* ‘Zebrinus’ leaves as experimental materials. By employing a combined analysis technique of non-targeted metabolomics and high-throughput transcriptomics, we systematically identify and screen core pigment metabolites and secondary metabolites related to stripe formation, and comprehensively explore differentially expressed genes in key pathways such as pigment synthesis. Through the correlation analysis of multi-omics data, this study aims to reveal the intrinsic regulatory mechanism of stripe formation in *M. sinensis* ‘Zebrinus’ leaves at both the molecular expression and metabolic accumulation levels, improve the theoretical system of stripe trait formation in *M. sinensis* ‘Zebrinus’, and ultimately provide important theoretical basis and technical support for the molecular genetic improvement of ornamental plant stripe traits and the targeted breeding of high-quality striped varieties.

## 2 Materials and methods

### 2.1 Material and collection place

**Figure 1:**
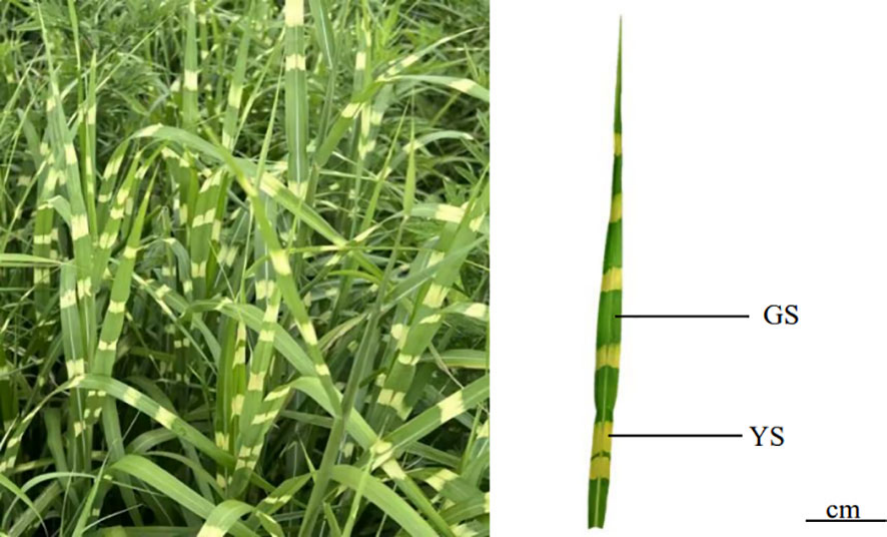
Morphology of different phenotypic leaves of *M. sinensis* ‘Zebrinus’. YS: Yellow area of variegated leaves; GS: Green area of variegated leaves.

The present study was conducted on two different leaf phenotypes [Yellow area of variegated leaves (YS) and Green area of variegated leaves (GS)] of *M. sinensis* ‘Zebrinus’ (**Figure 1**). They were collected on July 2, 2024, from 9:00 to 11:00 at the Yunnan Mountain Animal Husbandry Science and Technology Demonstration Park (103°16′–103°45′ E, 25°08′–25°37′ N). According to the 5-point sampling method was used to select 10 healthy plant clusters, and 6 top tender leaves were collected from each cluster. The isolated leaves were immediately placed in a precooled dry ice incubator (-78°C) to ensure anatomical separation of the yellow area of variegated leaves (YS) and green area of variegated leaves (GS) parts within 30 minutes. Each sample had three replicates, which were placed in 10 mL test tubes and stored in a -80°C refrigerator.

### 2.2 Metabolite extraction and ultra-performance liquid chromatography tandem mass spectrometry

Following vacuum freeze-drying in a freezer dryer (Scientz-100F, China), the experimental samples were ground into powder using a grinder (MM 400, Retsch). Then, 50 mg of sample powder were weighed and added with 1,200 μL of pre-cooled 70% methanol-water (Merck) internal standard extraction solution. After vortex-based processing on a vortex mixer (VORTEX-5, Kyllin-Bell) once every 30 min (30 s each, and 6 times totally), the obtained samples were centrifuged at 12,000 rpm for 3 min (5424R, Eppendorf). After that, with the supernatant aspirated (Biomek i5, Eppendorf), the sample was filtered through a 0.22 μm microporous membrane, and store in a brown injection vial. The ultra-performance liquid chromatography tandem mass spectrometry (LC-30A, Shimadzu) was performed according to previous method (Segers et al., 2019; Dettmer et al., 2007).

Raw data were subjected to the conversion into the mzXML format using ProteoWizard, followed by peak extraction and retention time alignment via the XCMS program. With the filtration of peaks with a missing rate > 50% in each sample group, the blank values were imputed using the K-nearest neighbor method; while the peak areas calibrated using the support vector regression method. After calibration and screening, metabolite identification of the peaks was conducted by searching the in-house database of the laboratory, integrated public databases, prediction databases, and via the metDNA approach. Criteria for the extraction of the eligible metabolites were: integrated identification score > 0.5 and coefficient of variation < 0.5 in quality control samples (Chen et al., 2009).

### 2.3 Transcriptomic analysis

In accordance with the protocol of the Trizol extraction kit (B511311, Shanghai Sangon), the total RNA was extracted from six *M. sinensis* ‘Zebrinus’ leaf samples. The intact total RNA was then subjected to processes such as mRNA isolation, fragmentation, double-stranded cDNA synthesis, cDNA fragment modification, magnetic bead purification, fragment sorting, and library amplification. Subsequent detection and quality control were performed to obtain the sequencing libraries suitable for the Illumina platform (Dewey and Li, 2011; Mortazavi et al., 2008). High-quality raw data was obtained through platform sequencing. FastQC was used to perform quality control on the raw sequencing data, removing sequencing adapters and low-quality bases. Trinity was employed to complete the assembly and optimize redundancy. The longest transcript of each gene was extracted as the representative reference sequence for unigene. With the assembled transcripts as reference sequences, the quality-controlled sequencing reads were aligned to the reference sequences using Bowtie2. Subsequently, the results of alignment were statistically analyzed using RSeQC. Finally, differential expression analysis was performed using DESeq, and significantly DEGs were identified based on the screening criteria of qValue < 0.05 and |FoldChange| > 2 (Zhang et al., 2023; Anders and Huber, 2010; Mao et al., 2005).

RNA-seq data is available in the NCBI database (http://www.ncbi.nlm.nih.gov/bioproject/1402042).

### 2.4 Quantitative real-time polymerase chain reaction

Total RNA was extracted from leaves with different phenotypes (Cao et al., 2023). After the determination of RNA purity using a nucleic acid and protein detector (Nano-600,China; A_260_/A_280_ ratio: 1.8~2.0), RNA integrity was verified by agarose gel electrophoresis (DYY-11, China). The qualified RNA was stored at -80°C for subsequent use.

Following the instructions of the reverse transcription kit (B639252, Shanghai Sangon), reverse transcription was performed in an RNase-free system using 2 μg of total RNA as the template. The reaction program was set as 5 min at 25°C, 15 min at 55°C, and 5 min at 85°C. The synthesized cDNA was diluted 10-fold with RNase-free water, and then stored at 4°C or at -20°C for short-term use or long-term preservation, respectively. Based on the instruction manual of SGExcel Universal SYBR qRT-PCR Mix (B532958, Shanghai Sangon) for the detailed reaction system and procedure, the qRT-PCR reactions were carried out on a qRT-PCR instrument using the SYBR Green I method. Each sample was set up with three biological replicates, using template-free water as the negative control. Genes with relatively stable expression levels and no significant differences between YS and GS were selected as internal reference genes to correct the sample loading (internal reference gene primer F: TTGGGTTTGTTAGGGGCCA; R: GGCTTTCAGAGATGCACATGA). Eventually, the relative expression levels of target genes were calculated by the 2^−ΔΔCt^ method (Zhao LQ, et al., 2024).

### 2.5 Data processing and statistical analysis

In our study, statistical analysis of all data was completed in Excel. One-way analysis of variance and duncan’s new multiple range test were performed for multiple comparisons of data via SPSS 27.0 software. The functional enrichment analysis and generation of heatmaps for metabolomic and transcriptomic data were conducted in Metware Cloud Platform (https://cloud.metware.cn). The co-enrichment analysis diagram for metabolomics and transcriptomics was plotted using the Metware Cloud Platform. Data were organized according to the platform’s data input requirements, and corresponding functions were selected in the cloud tool to upload and analyze the data. Based on the enriched pathways, the correlation between differential genes and metabolites was calculated, and the data were organized to generate a correlation network diagram using Cytoscape.

## 3 Results

### 3.1 Metabolomic differential analysis of different phenotypic leaves

**Figure 2:**
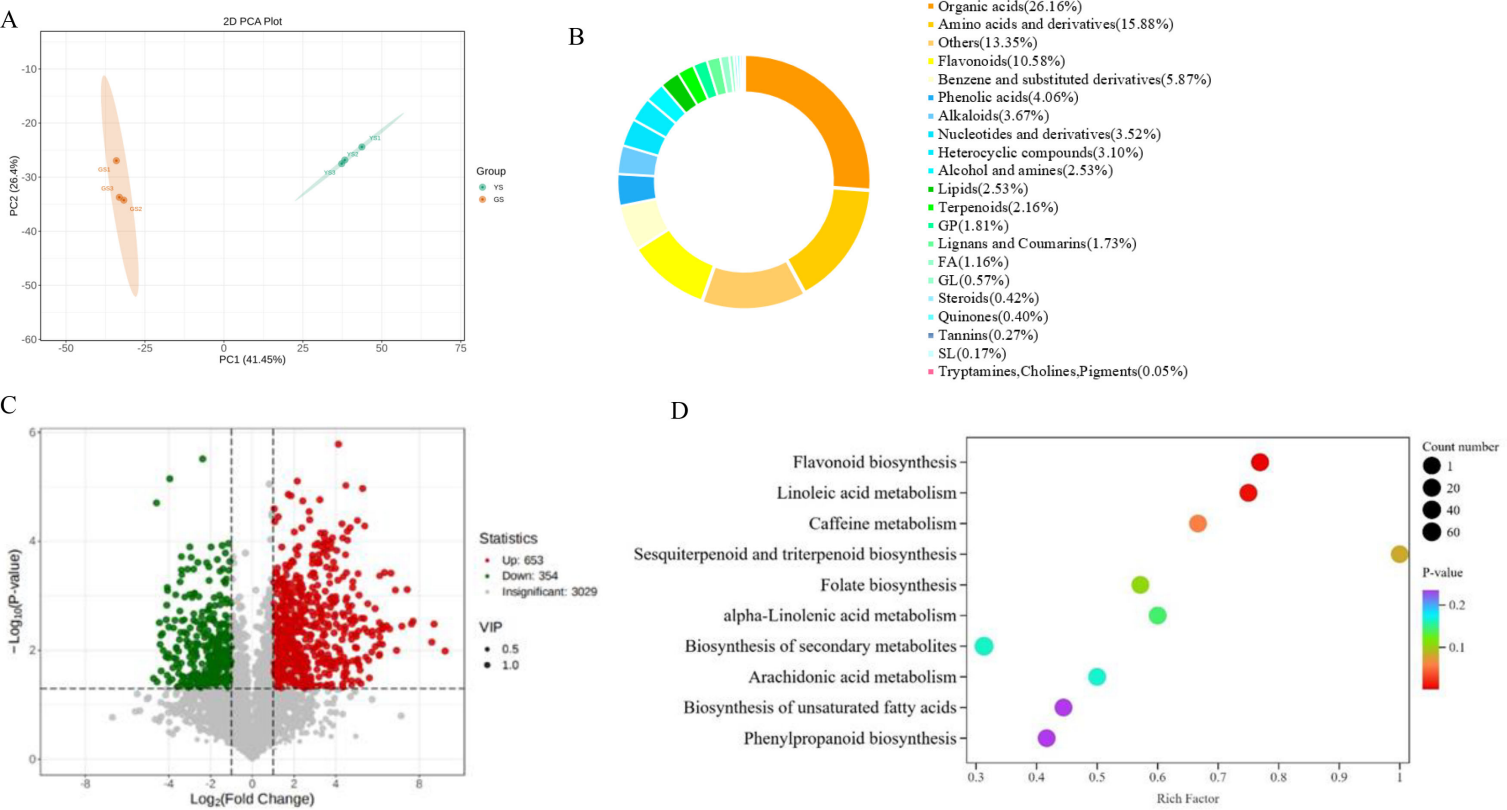
Differential analysis of metabolites. **(A)** Principal component analysis score plot of different phenotypes; **(B)** Metabolite classification and proportion diagram; **(C)** Volcano plot of differential metabolites; **(D)** Enrichment analysis of the top-10 KEGG bubble plot for pathway.

Principal component analysis (PCA) was performed on all samples to clarify the overall inter-group metabolomic differences and the intra-group degree of variation among samples. The PCA results (**Figure 2A**) revealed a distinct inter-group separation between YS and GS in the score plot, while samples within each group were tightly clustered without obvious separation. Collectively, these results suggested good biological reproducibility of samples in each group; high consistency of metabolomic data among intra-group samples; as well as clear and significant metabolomic difference between YS and GS.

Non-targeted metabolomic analysis further identified 4,036 metabolites (**Supplementary Table S1**), which were categorized into 21 metabolic classes. Among these, organic acids accounted for the highest proportion (26.16%), followed by amino acids and their derivatives, while flavonoids (excluding the “Others” category) ranked third (**Figure 2B**). Screening for differential metabolites between groups (**Figure 2C**) yielded 653 significantly upregulated metabolites and 354 significantly downregulated metabolites in the YS group compared with the GS group. Kyoto Encyclopedia of Genes and Genomes (KEGG) pathway enrichment on these differential metabolites indicated significant enrichment of inter-group differential metabolites in the flavonoid biosynthesis pathway (**Figure 2D**).

Additionally, hierarchical clustering analysis was performed on the differential metabolites (**Supplementary Figure S1**; **Supplementary Table S2**) to further elucidate the characteristics of metabolic differences between different phenotypic leaves. Consequently, metabolic classes such as amino acids and their derivatives, organic acids, as well as benzene and its derivatives were significantly enriched in GS; in contrast, flavonoid compounds were specifically and significantly enriched in YS. Altogether, these data highlighted potentially close association of the significant enhancement of flavonoid metabolic pathway flux with the formation of leaf variegation phenotypes.

### 3.2 Metabolites associated with leaf variegation

**Figure 3:**
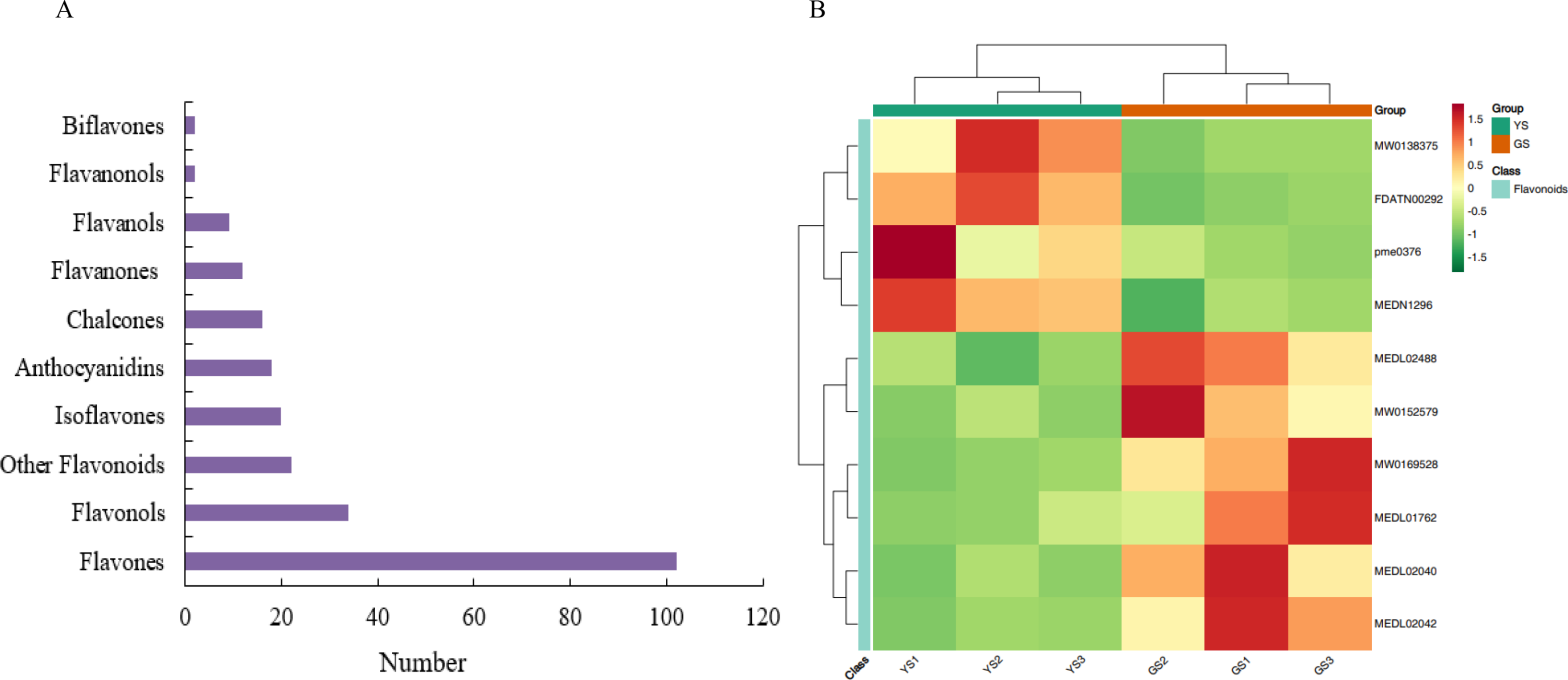
Differential analysis of flavonoids. **(A)** Secondary classification diagram of flavonoids; **(B)** Heatmap of expression patterns of 10 flavonoid metabolites.

By integrating KEGG pathway enrichment analysis and clustering analysis, significant enrichment of differential metabolites between YS and GS was observed in the flavonoid biosynthesis pathway. Accordingly, we continued to carry out a secondary classification and statistical analysis of flavonoid metabolites (**Figure 3A**). As a result, among the secondary classifications of flavonoids, flavones were the most abundant, followed by flavonols. Subsequently, 10 flavonoid metabolites with the most significant differences between groups were selected for expression pattern heatmap analysis (**Figure 3B**; **Supplementary Table S3**) to identify the flavonoid metabolites associated the most closely with the leaf variegation phenotype. Neohesperidin (MW0138375), taxifolin (FDATN00292), naringenin (pme0376), and xanthohumol (MEDN1296) exhibited significantly higher expressions in the YS group than those in the GS group. In contrast, quercetin (MEDL02488), leucodelphidin (MW0152579), naringin (MW0169528), sakuranetin (MEDL01762), gallocatechin (MEDL02040), and cianidanol (MEDL02042) had obviously lower expressions in the YS group compared to the GS group. With regard to the above, the altered expression level of naringenin might be one of the key metabolites regulating the formation of yellow leaf variegation.

### 3.3 NR annotation analysis

**Figure 4:**
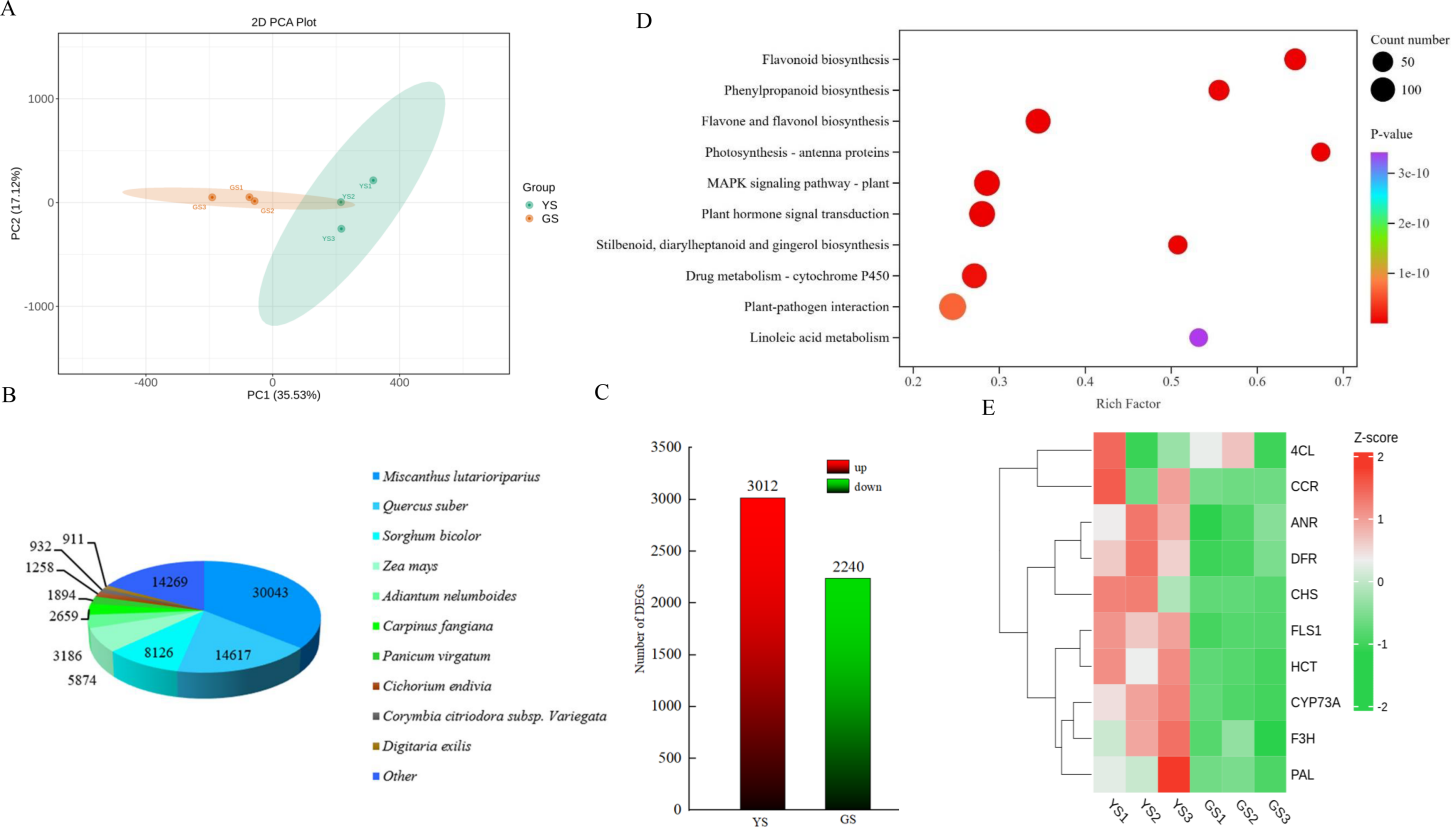
Transcriptome sequencing analysis. **(A)** Principal component analysis score plot of different phenotypes; **(B)** Pie chart of homologous species distribution; **(C)** Bar chart of differentially expressed genes; **(D)** The functional enrichment bubble plots of top-10 differentially expressed genes; **(E)** Cluster analysis of 10 genes. *4CL*, 4-coumarate--CoA ligase; *CCR*, Cinnamoyl-CoA reductase; *ANR*, Anthocyanidin reductase; *DFR*, Dihydroflavonol 4-reductase; *CHS*, Chalcone synthase; *FLS1*, Flavonol synthase 1; *HCT*, Hydroxycinnamoyl-CoA shikimate/quinate hydroxy cinnamoyl transferase; *CYP73A*, Trans-cinnamate 4-monooxygenase; *F3H*, Flavonoid 3-hydroxylase; *PAL*, Phenylalanine ammonia-lyase.

Transcriptome sequences were obtained through assembly (**Table 1**). To evaluate the quality of the assembly and the coverage of the species database, the sequences were aligned with the Non-Redundant Protein Sequence Database (NR) (**Figure 4B**). It was observed that the transcript sequences of *M. sinensis* ‘Zebrinus’ showed the highest number of matches with *Miscanthus lutarioriparius* (30,043), followed by *Quercus suber* (14,617), *Sorghum bicolor* (8,126), *Zea mays* (5,874), *Adiantum nelumboides* (3,186), *Carpinus fangiana* (2,659), *Panicum virgatum* (1,894), *Cichorium endivia* (1,258), *Corymbia citriodora subsp*. variegata (932), and *Digitaria exilis* (911). Notably, *M. lutarioriparius*, *S. bicolor*, *Z. mays*, *P. virgatum*, and *D. exilis* all belong to the *Poaceae* family, sharing a closer taxonomic relationship with *M. sinensis* ‘Zebrinus’. Therefore, the transcript sequences of *M. sinensis* ‘Zebrinus’ obtained in this study were highly reliable, which could support subsequent functional gene mining and molecular mechanism research.

**Table 1:** Statistics of assembly results.

Type	Number	N50	N90	Average length
Transcript	349613	1269	345	828.49
Unigene	139118	1074	260	663.19

### 3.4 Analysis of DEGs in different phenotypic leaves

By conducting PCA analysis on the distribution characteristics of transcriptome data dimensions for samples from the YS group and GS group, the results indicated good intra-group reproducibility and significant inter-group differences (**Figure 4A**). RNA-seq technology was employed to screen DEGs between YS and GS, resulting in the identification of 5,252 DEGs totally. Among these, 3,012 genes were significantly upregulated and 2,240 genes were obviously downregulated (**Figure 4C**). Subsequent enrichment analysis revealed significant enrichment of these DEGs in the flavonoid biosynthesis and phenylpropanoid biosynthesis pathways. It could be speculated that the formation of leaf variegation might be regulated primarily by the flavonoid biosynthesis and phenylpropanoid biosynthesis pathways (**Figure 4D**). It was inferred that differential expression of key genes in these pathways might provide essential molecular foundation for leaf phenotypic differentiation.

### 3.5 qRT-PCR analysis

**Figure 5:**
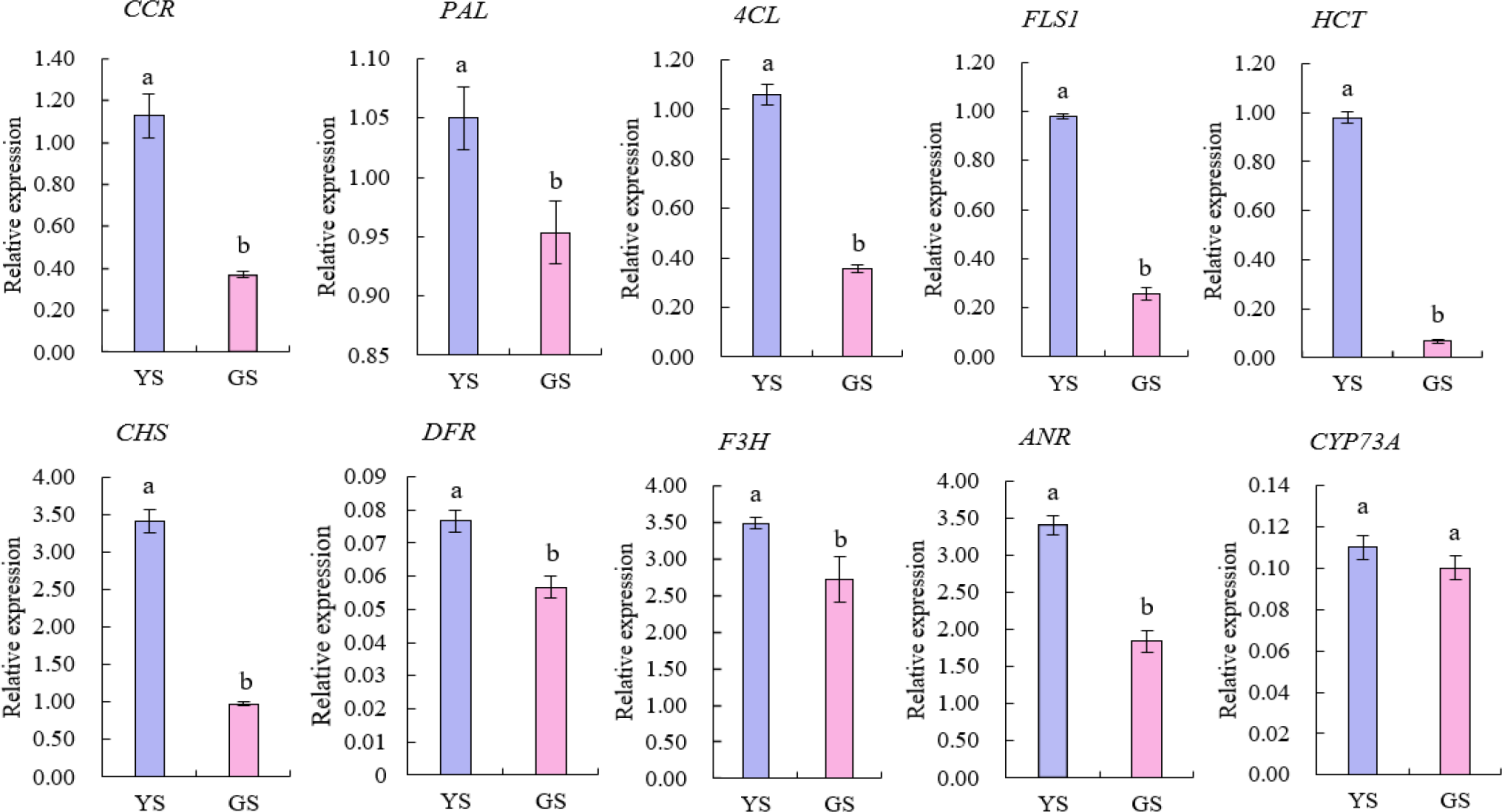
Relative expression levels of differentially expressed genes. Data are shown as mean ± standard error based on three replicates. Different alphabets indicate significant differences at *P* < 0.05 according to the Duncan’s multiple range test.

As presented in **Figure 4E** and **Supplementary Table S4**, **5** candidate genes with significantly differential expression in YS were selected respectively from the flavonoid biosynthesis and phenylpropanoid biosynthesis pathways. According to the verification and analysis of their expression levels by qRT-PCR (**Figure 5**), the expression levels of the other 9 candidate genes in YS were significantly higher than those in GS (all *P<0.05*), except for the gene *CYP73A* without no significant statistical difference in expression level between YS and GS (*P>0.05*). Further analysis revealed that the overall expression abundances of *CHS*, *F3H* and *ANR* were the highest among the two phenotypes. Specifically, the expression level of *CHS* gene in YS was approximately 3 times that in GS, exhibiting an extremely significant phenotype-specific difference. Collectively, *CHS* gene might be a key candidate gene regulating the yellowing of leaf variegation in *M. sinensis* ‘Zebrinus’.

### 3.6 Integrated metabolomic and transcriptomic analysis

**Figure 6:**
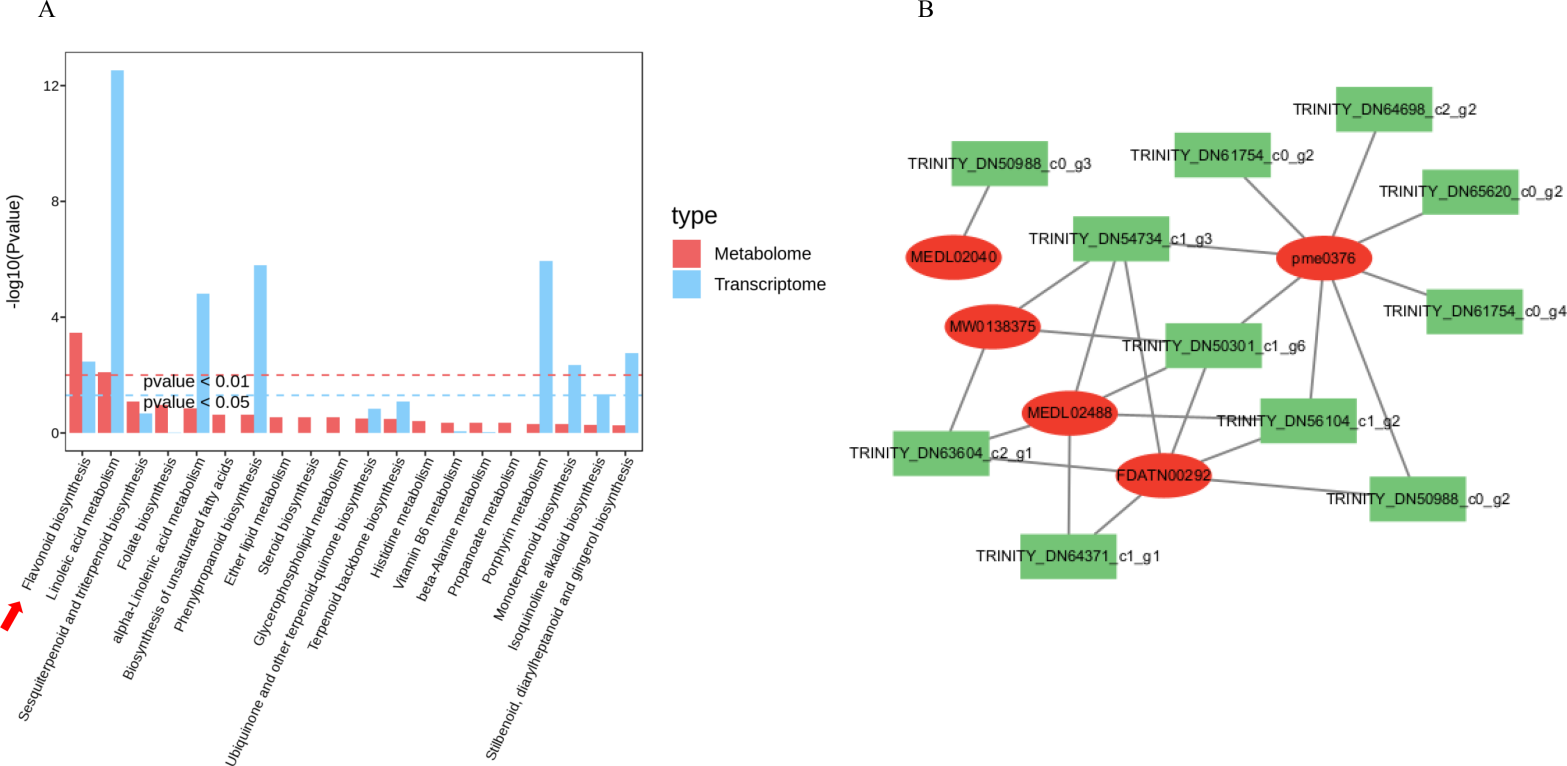
Integrated metabolomic and transcriptomic analysis. **(A)** Co-enrichment analysis diagram of the top-20 pathways; **(B)** Correlation network diagram between differential metabolites and differentially expressed genes.

Subsequently, metabolomic and transcriptomic data were integrated to clarify the core regulatory pathway underlying leaf variegation formation in *M. sinensis* ‘Zebrinus’. The results revealed that the flavonoid biosynthesis was the primary pathway regulating leaf variegation formation (**Figure 6A**). On this basis, a correlation network was further constructed and analyzed for key flavonoid metabolites and DEGs involved in leaf variegation formation (**Figure 6B**). Consequently, the highest correlation coefficient was observed between chalcone synthase gene *CHS* (TRINITY_DN65620_c0_g2) and the metabolite naringenin (pme0376), reaching 0.96 (**Supplementary Table S5**). It is speculated that the *CHS* gene may be a key regulatory gene for the biosynthesis of naringenin in the leaves of *M. sinensis* ’Zebrinus’.

## 4 Discussion

In general, the phenotypic variation of plant leaf color is a complex biological process regulated by the synergy of multiple factors. In addition to being dynamically regulated by external environmental factors such as light and temperature, it may has an intimate association with changes in the types and contents of differential metabolites in plants (Wu et al., 2020). In this study, GS and YS, two distinct leaf color phenotypes of *M. sinensis* ‘Zebrinus’, were used for subsequent investigations. This study achieved a systematic research of the metabolic regulatory mechanism underlying leaf variegation in *M. sinensis* ‘Zebrinus’ by integrating metabolomics, transcriptomics techniques, and combining with the KEGG pathway co-enrichment analysis.

Screening and enrichment analysis of differential metabolites in the two phenotypic leaves revealed significant enrichment of these differential metabolite in the flavonoid biosynthesis pathway. Furthermore, flavonoid compounds were the key metabolites involved in the formation of yellow leaf variegation in *M. sinensis* ‘Zebrinus’, with significant accumulation of naringenin in YS. Existing studies have confirmed the significant regulatory roles of the accumulation level and component differences of flavonoid compounds in driving the phenotypic differentiation of plant leaf/flower color (Shen et al., 2018; Zhang et al., 2022). Shi et al. found that the synergistic accumulation of flavones and flavonols can significantly promote the leaf yellowing process of *G. biloba* leaves (Shi et al., 2012). These substances can also enhance the color intensity and vividness of autumn leaf color in *Cotinus coggygria* through copigmentation. Meanwhile, 196 flavonoid-related metabolites were detected in the study on the flavonoid metabolome of *Cymbidium sinense* leaves, among which 42 metabolites revealed significant content differences among different leaf color phenotypes (Gao et al., 2020). Similarly, in another study on the petals of *Gossypium hirsutum*, flavonoid compounds had direct relationship with the phenotypic formation of yellow petals (Tan et al., 2018). By systematically analyzing four major plant pigment substances (chlorophyll, carotenoids, flavonoids, and anthocyanins) quantitatively, He et al. clarified that flavonoid content was a key determinant of the yellow peel phenotype of *Cucumis sativus* (He et al., 2023). Consistent with the conclusions of previous studies mentioned above, in our study, the formation of yellow leaf variegation in *M. sinensis* ‘Zebrinus’ was regulated dominantly by flavonoid compounds. It further confirmed that the specific accumulation of flavonoid compounds was the core reason for the formation of yellow variegation in *M. sinensis* ‘Zebrinus’, and naringenin might be the key functional metabolite mediating the formation of this leaf color phenotype. This conclusion aligns with the yellowing of “Rougui” tea leaves caused by naringenin (Wang et al., 2020), further confirming that naringenin is the primary metabolite responsible for the yellow variegation on the leaves.

In this study, comparative transcriptome analysis on GS and YS tissues of *M. sinensis* ‘Zebrinus’ revealed significant enrichment of the DEGs in the flavonoid biosynthesis and phenylpropanoid biosynthesis pathways. It is in line with the general rule that plant leaf variegation is often accompanied by secondary metabolic pathway remodeling (Zhang et al., 2025; Deng and Lu, 2017), confirming that plant leaf color differentiation may be determined by expression variation of genes related to pigment synthesis and metabolism (Song et al., 2017). Flavonoids is a widely existing water-soluble pigment in plants (Tsukasa, 2000) that directly affect leaf phenotypes through their types and contents, and also serve as important secondary metabolites in plants (Zhao et al., 2025). The regulation of this pathway relies on the synergistic action of key enzyme genes such as *CHS* and *F3’H*, whose changes in expressions can directly alter the content of flavonoids in leaves, thereby triggering leaf variegation (Zhang et al., 2025; Wang Y, et al., 2025). *CHS* is a rate-limiting enzyme in flavonoid synthesis, and its high expression can strongly drive the metabolic flux toward flavonoid synthesis, further promoting the directional accumulation of flavonoid pigments in plants. In this study, the expression level of *CHS* in YS was significantly higher than that in GS. Meanwhile, previous physiological determination results have revealed higher total flavonoid content in YS than that in GS. Therefore, the differential activation of the flavonoid biosynthesis pathway can act as the direct driver of the formation of the YS phenotype in *M. sinensis* ‘Zebrinus’ by regulating the synthesis and accumulation of yellow pigments. A similar regulatory pattern has also been documented in *Liquidambar formosana*, where the upregulation of flavonoid synthesis genes during color transition might induce changes in leaf color (Lai et al., 2022; Li et al., 2024).

The phenylpropanoid pathway serves as the central upstream pathway of secondary metabolism in plants. Its core enzyme, *PAL*, can catalyze the conversion of phenylalanine to cinnamic acid, presenting the common precursor for downstream pathways such as flavonoid biosynthesis, forming the “material basis” for pigment synthesis (Wang Y, et al., 2025). The activity of this pathway can determine the efficiency of precursor supply for downstream pigment synthesis directly, thereby affecting the final pigment accumulation level (Wang SQ, et al., 2025). In this study, both the expression levels of *PAL* and *C4H* were significantly higher in YS than that in GS. It highlights a specific activation of the phenylpropanoid pathway in YS tissues. The synergistic high expression of *PAL* and *C4H* results in a more sufficient supply of cinnamic acid precursors for downstream flavonoid pathway. It leads to the formation of a metabolic flux synergy effect characterized by “increased upstream donors - enhanced downstream pigment synthesis”. In contrast, in GS tissues, both *PAL* and *C4H* were lowly expressed, indicating insufficient supply of flavonoid synthesis precursors and low accumulation of yellow pigments, ultimately maintaining the chlorophyll-dominated green phenotype. Collectively, the proposed mechanism conforms to the general rule of “upstream pathways supporting downstream product synthesis” in plant secondary metabolism (Wang Y, et al., 2025).

Our future direction of research may involve the investigation of the regulatory relationship between naringenin and *CHS*. We may continue to carry out *CHS* gene overexpression/silencing experiments to clarify the specific regulatory efficiency of *CHS* on naringenin biosynthesis. Meanwhile, we will investigate whether *MYB*, *bHLH* and other transcription factors can bind to *CHS*, thereby co-regulating the “gene-metabolism” pathway in leaf variegation of *M. sinensis* ‘Zebrinus’.

## 5 Conclusions

In conclusion, this study through a combined metabolomic and transcriptomic analysis, concluded that the main metabolite affecting the formation of leaf variegation on *M. sinensis* ‘Zebrinus’ is naringenin, and CHS is the key factor regulating its leaf variegation formation. Furthermore, naringenin and CHS exhibit a high correlation, leading to the preliminary speculation that the expression of the *CHS* gene is closely intrinsically related to the metabolic accumulation of naringenin. The *CHS* gene may participate in the formation process of yellow leaf variegation by mediating the synthesis and distribution of naringenin substances in plants. This study reveals the molecular basis of leaf variegation formation in *M. sinensis* ‘Zebrinus’, fills the gap in the research on the mechanism of leaf variegation formation in gramineae plants, and provides an important theoretical reference for the formation mechanism of leaf variegation phenotypes in other herbaceous plants.

## Data Availability

The original contributions presented in the study are included in the article/**Supplementary Material**. Further inquiries can be directed to the corresponding author.
