# Supplementary figures and images for: Integrated metabolomic and transcriptomic analysis of the mechanism underlying leaf variegation in Miscanthus sinensis ‘Zebrinus’
Source: Front Plant Sci. 2026 Feb 11;17:1748715. doi: 10.3389/fpls.2026.1748715 (PMC12932611; doi:10.3389/fpls.2026.1748715)

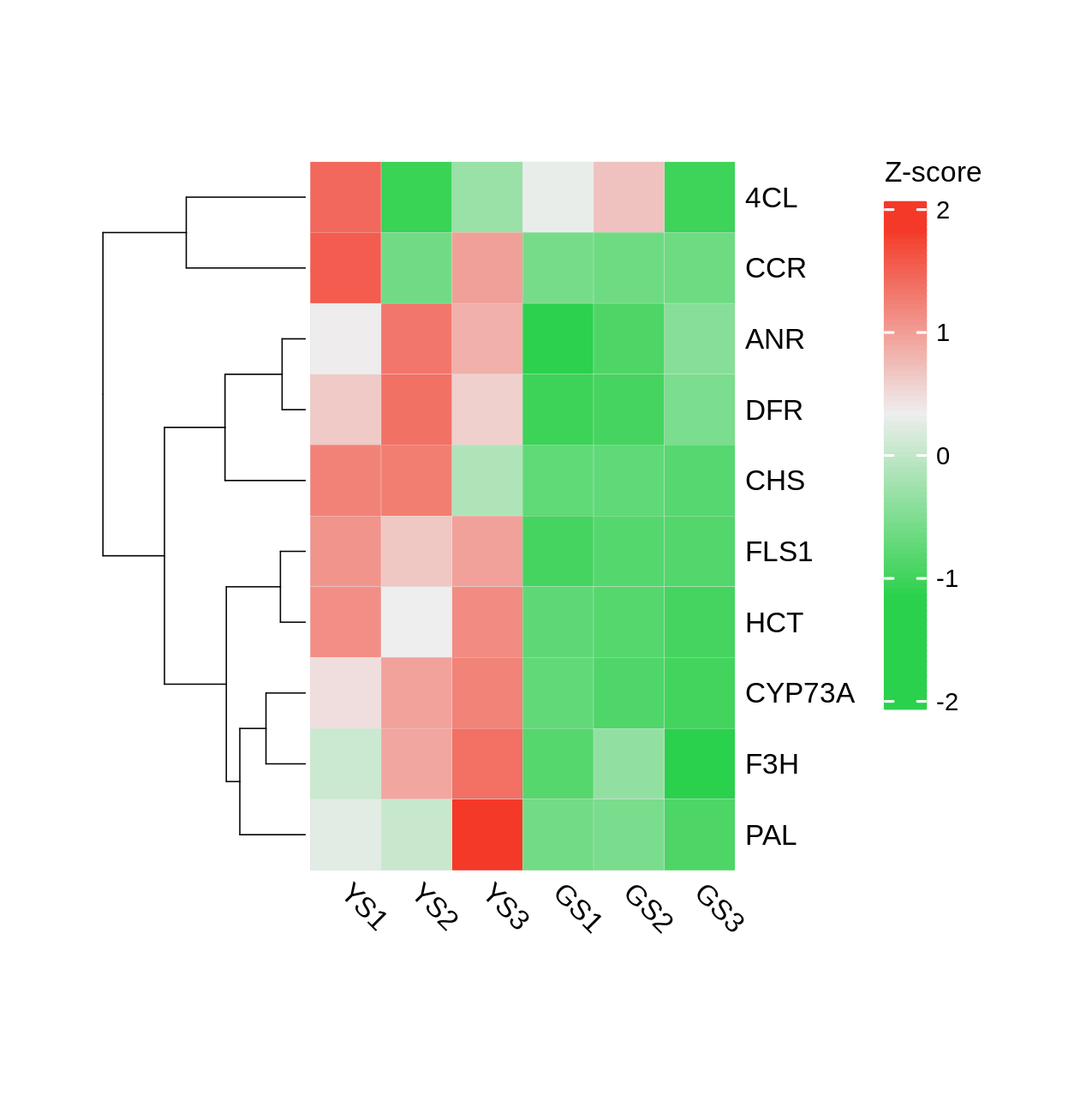

Supplement: Supplementary file 1 [file Image1.tiff]

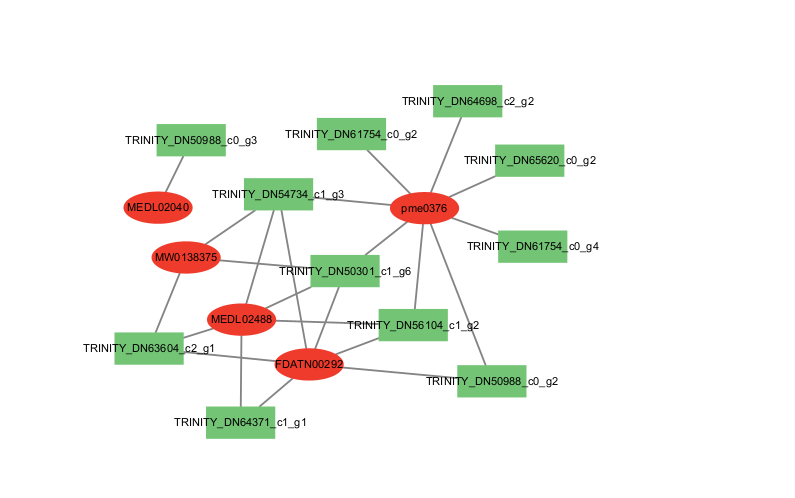

Supplement: Supplementary file 2 [file Image2.png]

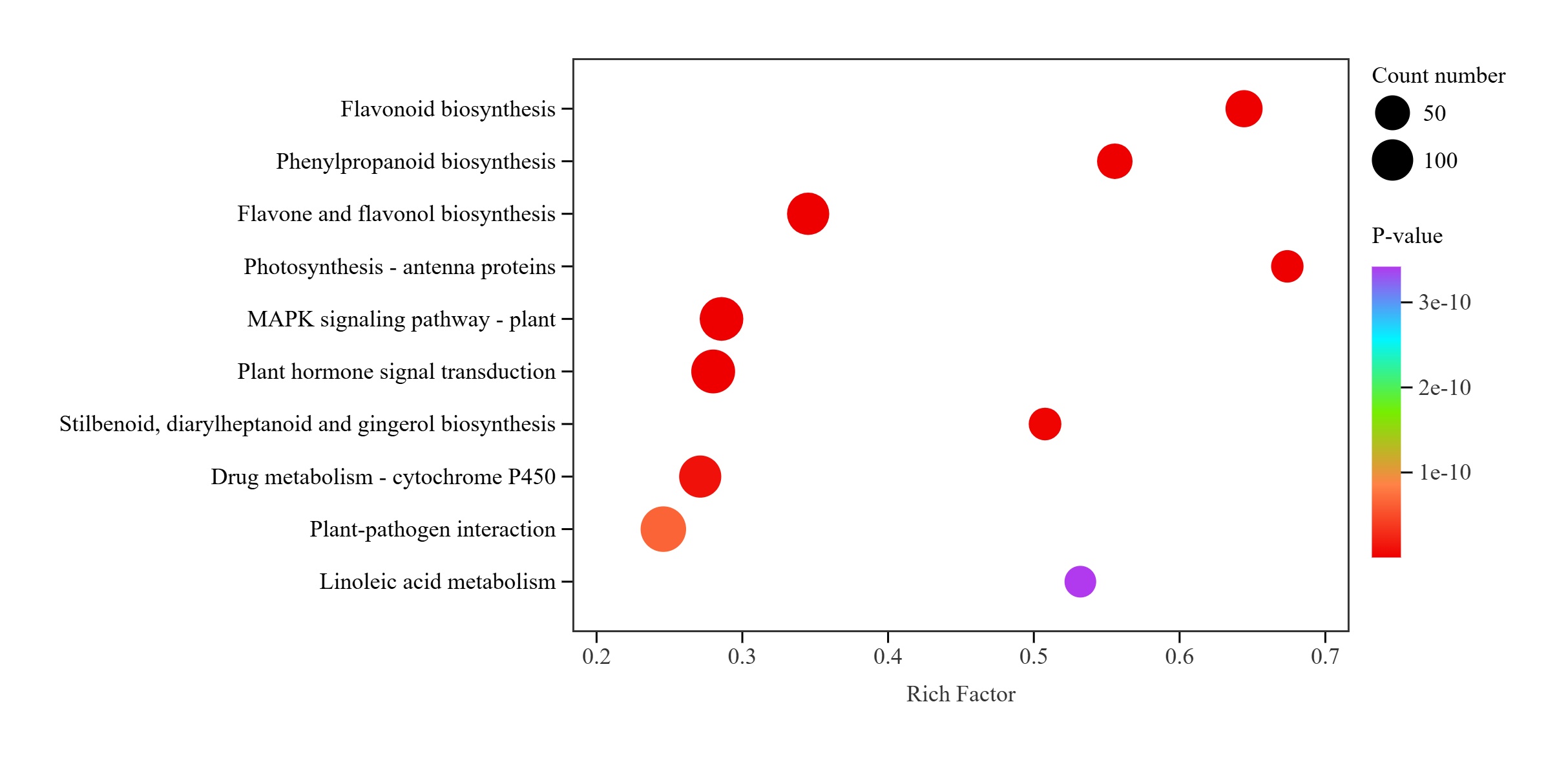

Supplement: Supplementary file 3 [file Image3.jpeg]

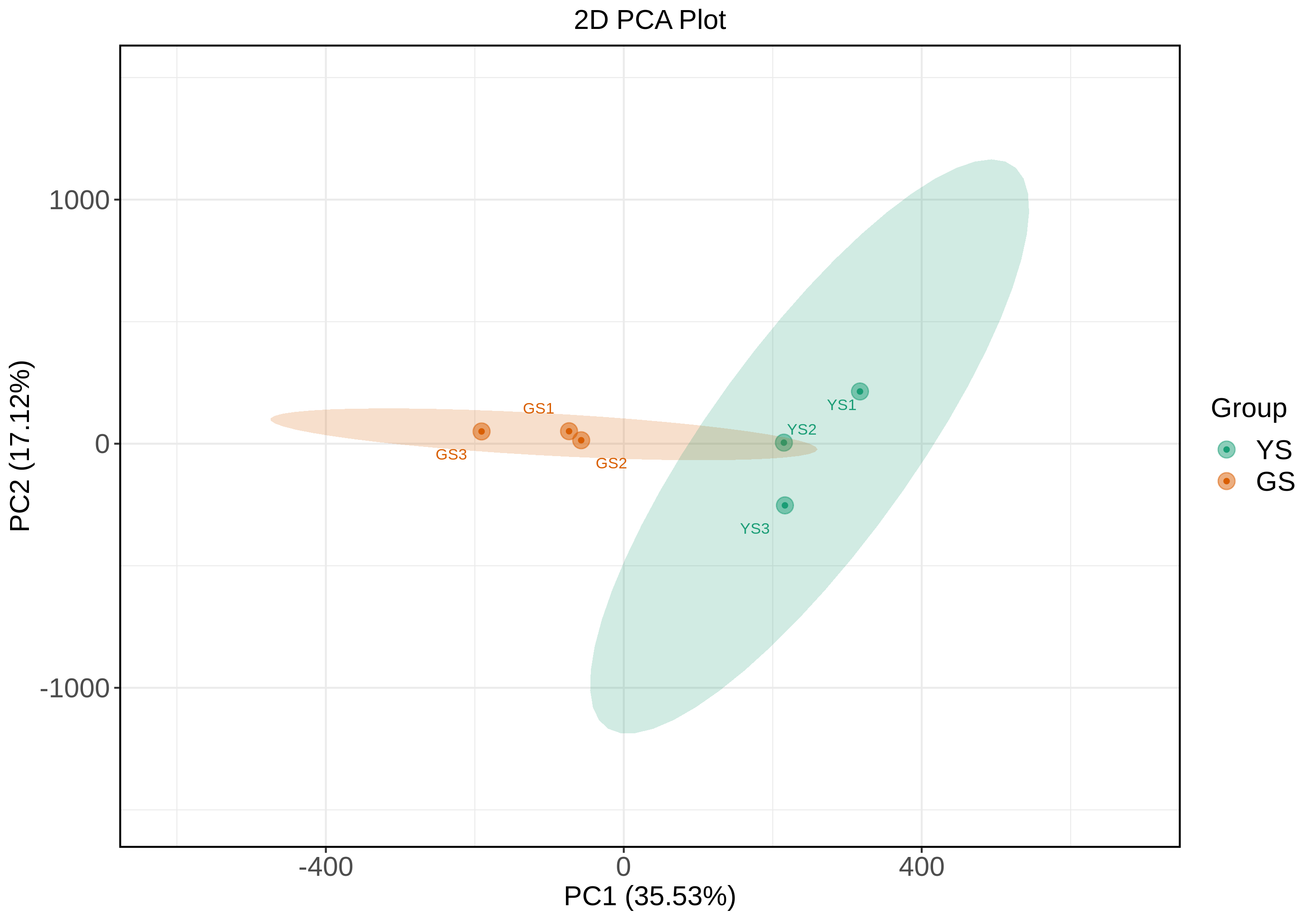

Supplement: Supplementary file 4 [file Image4.png]

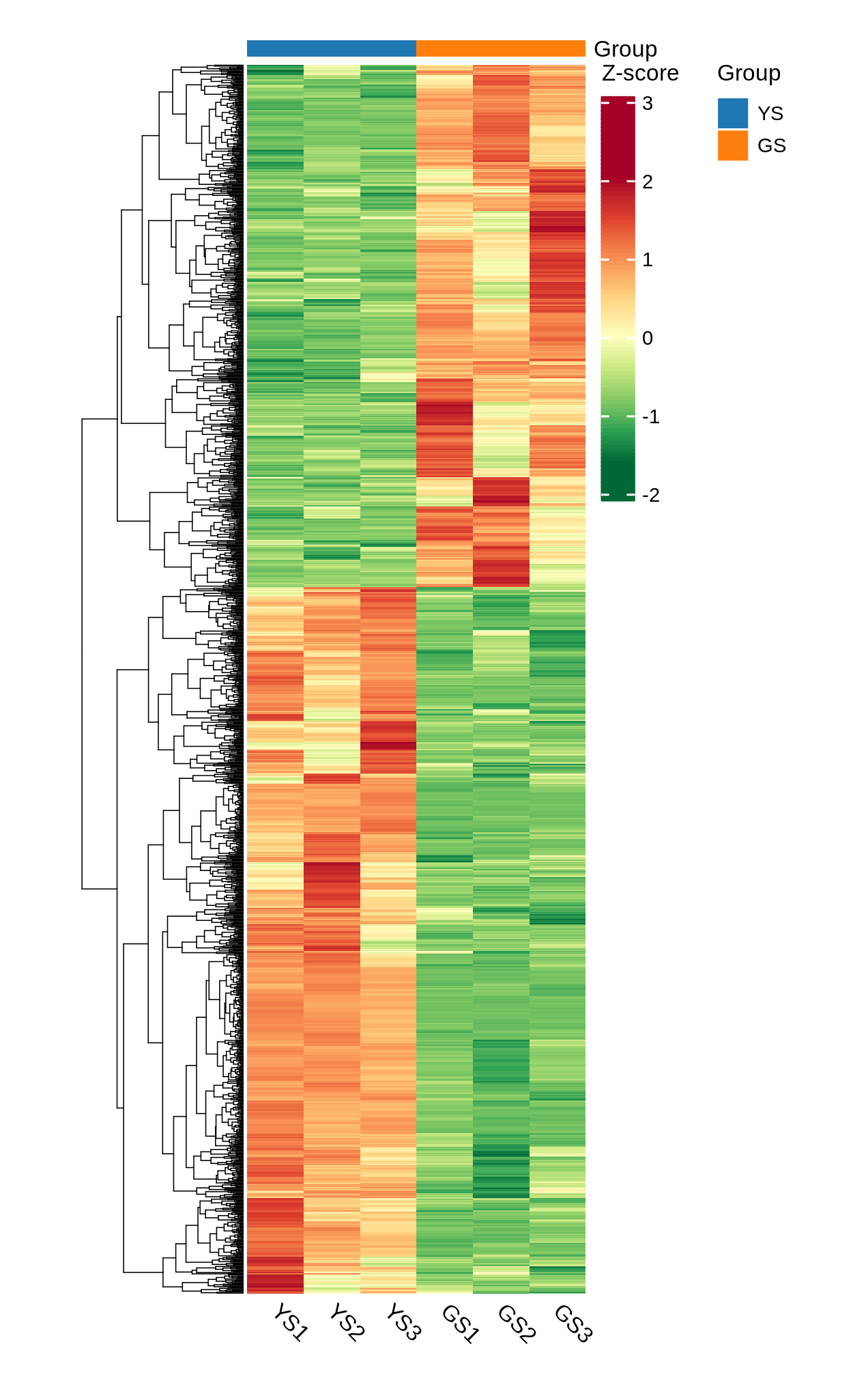

Supplement: Supplementary file 5 [file Image5.png]

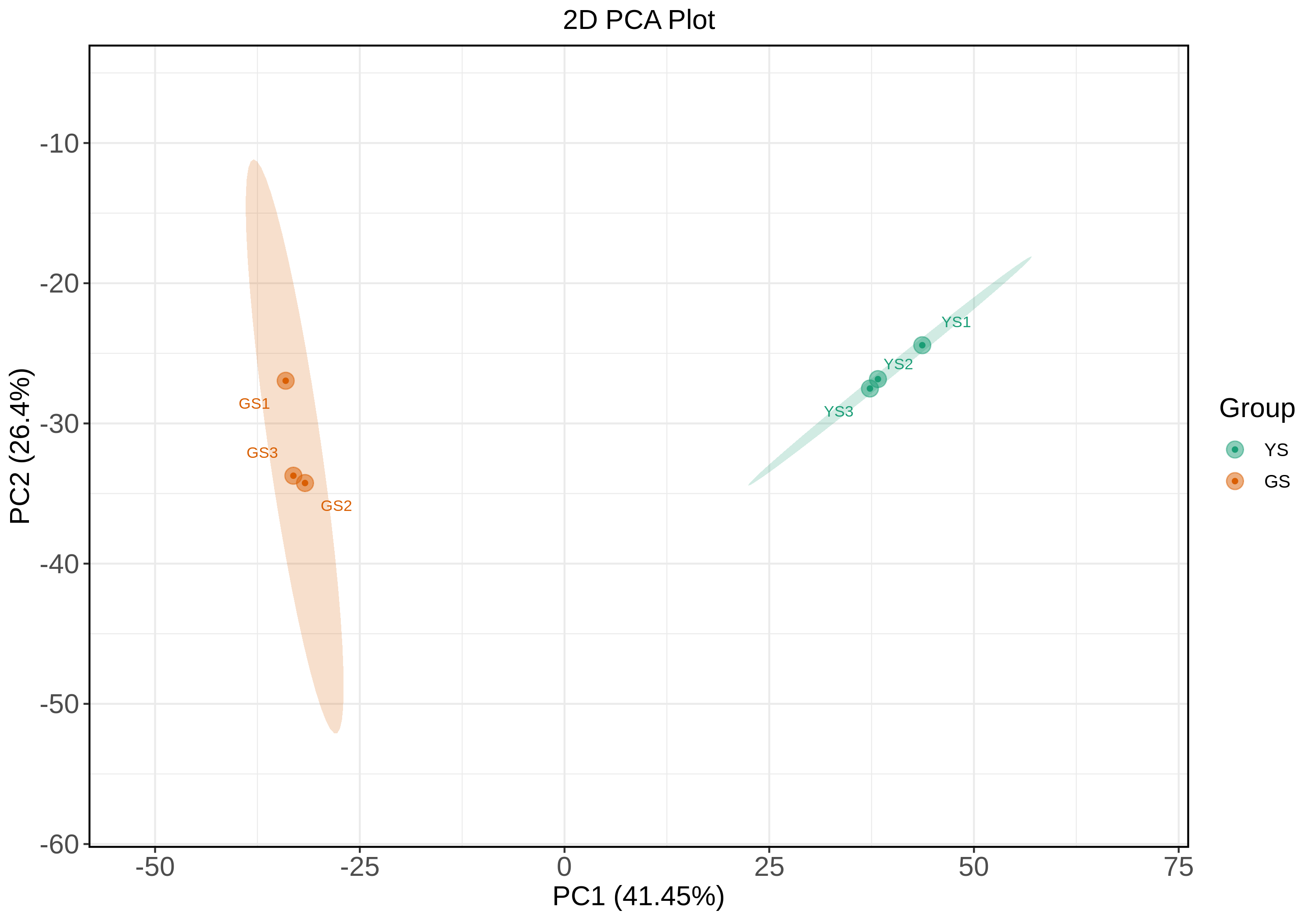

Supplement: Supplementary file 6 [file Image6.png]

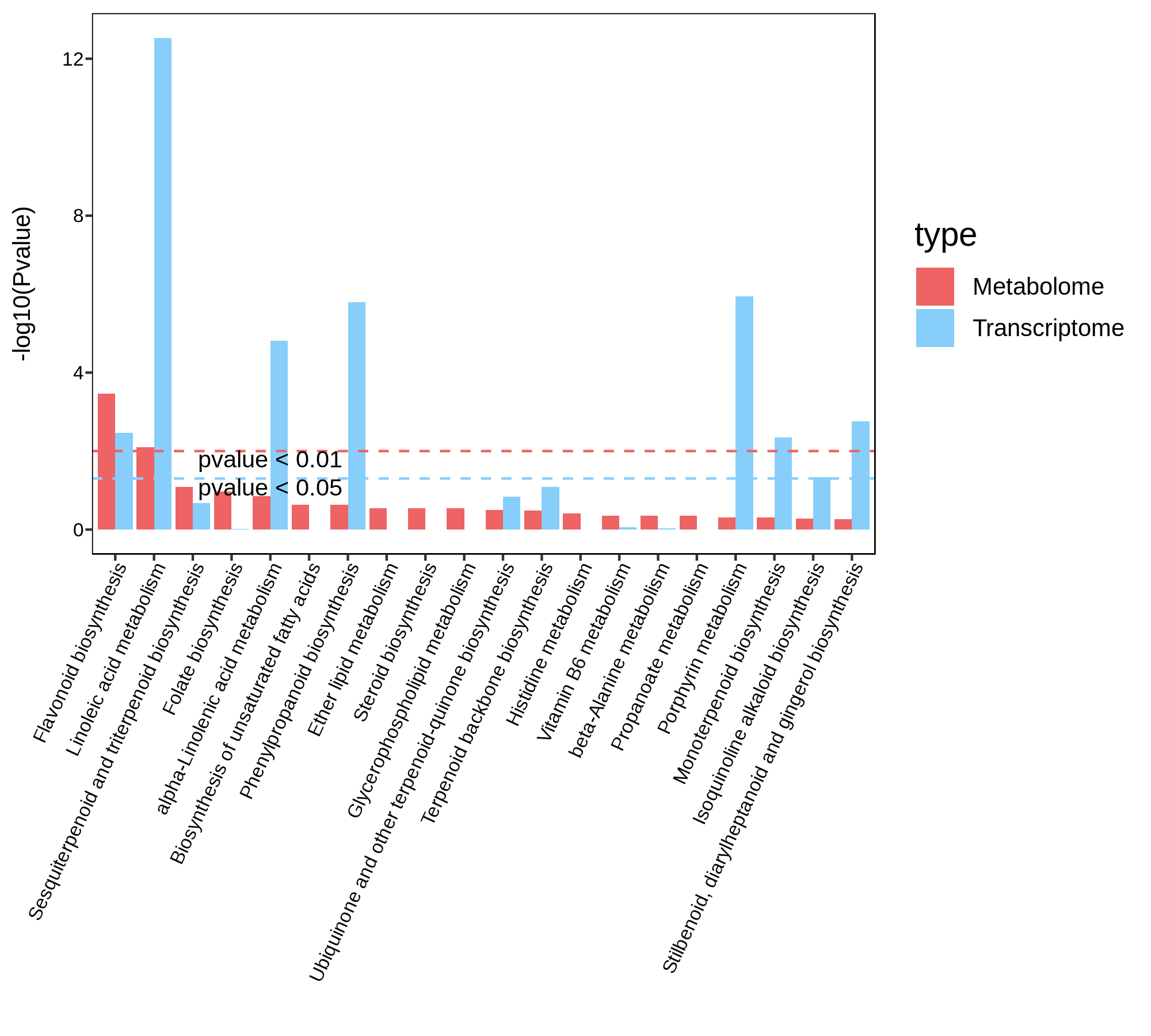

Supplement: Supplementary file 7 [file Image7.png]

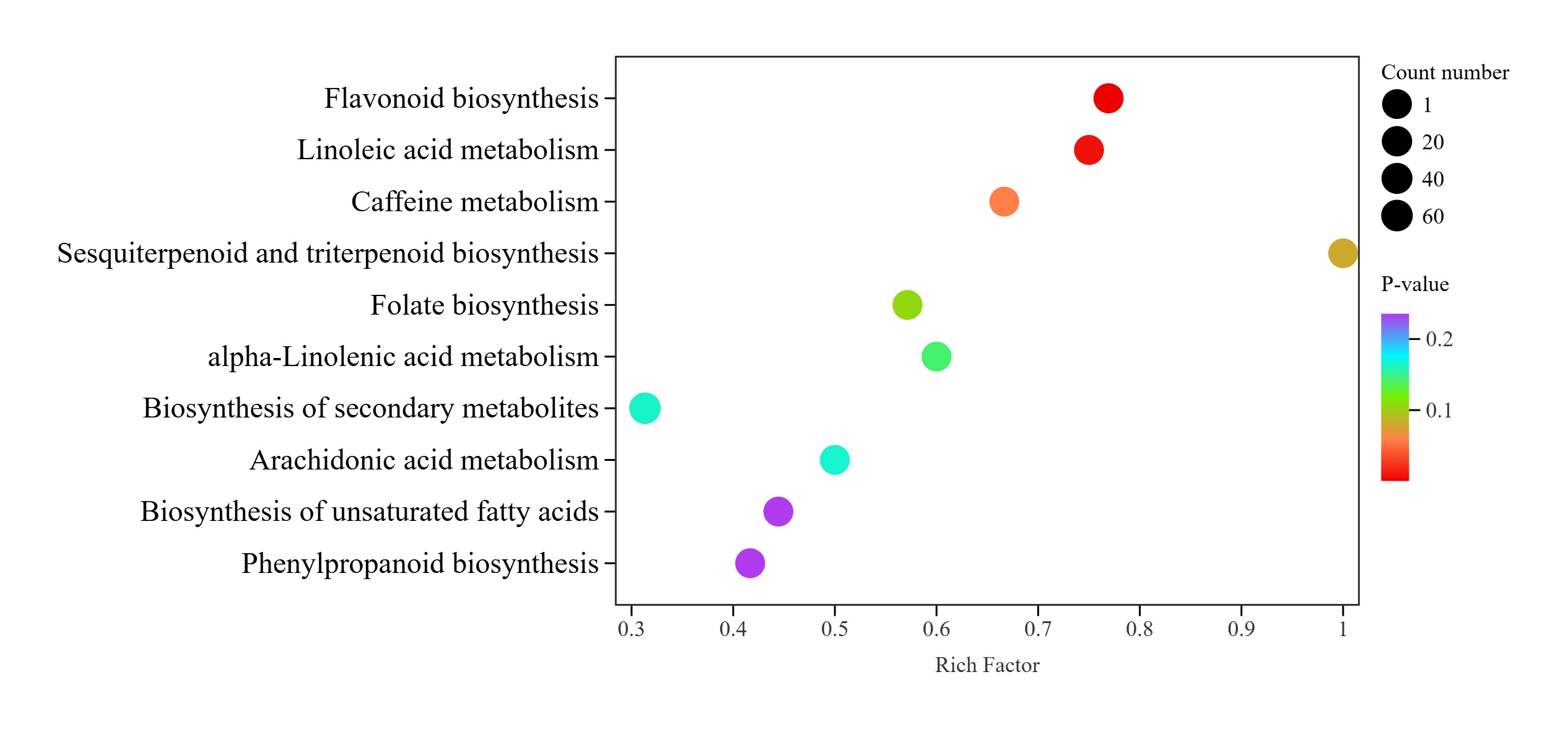

Supplement: Supplementary file 8 [file Image8.jpeg]

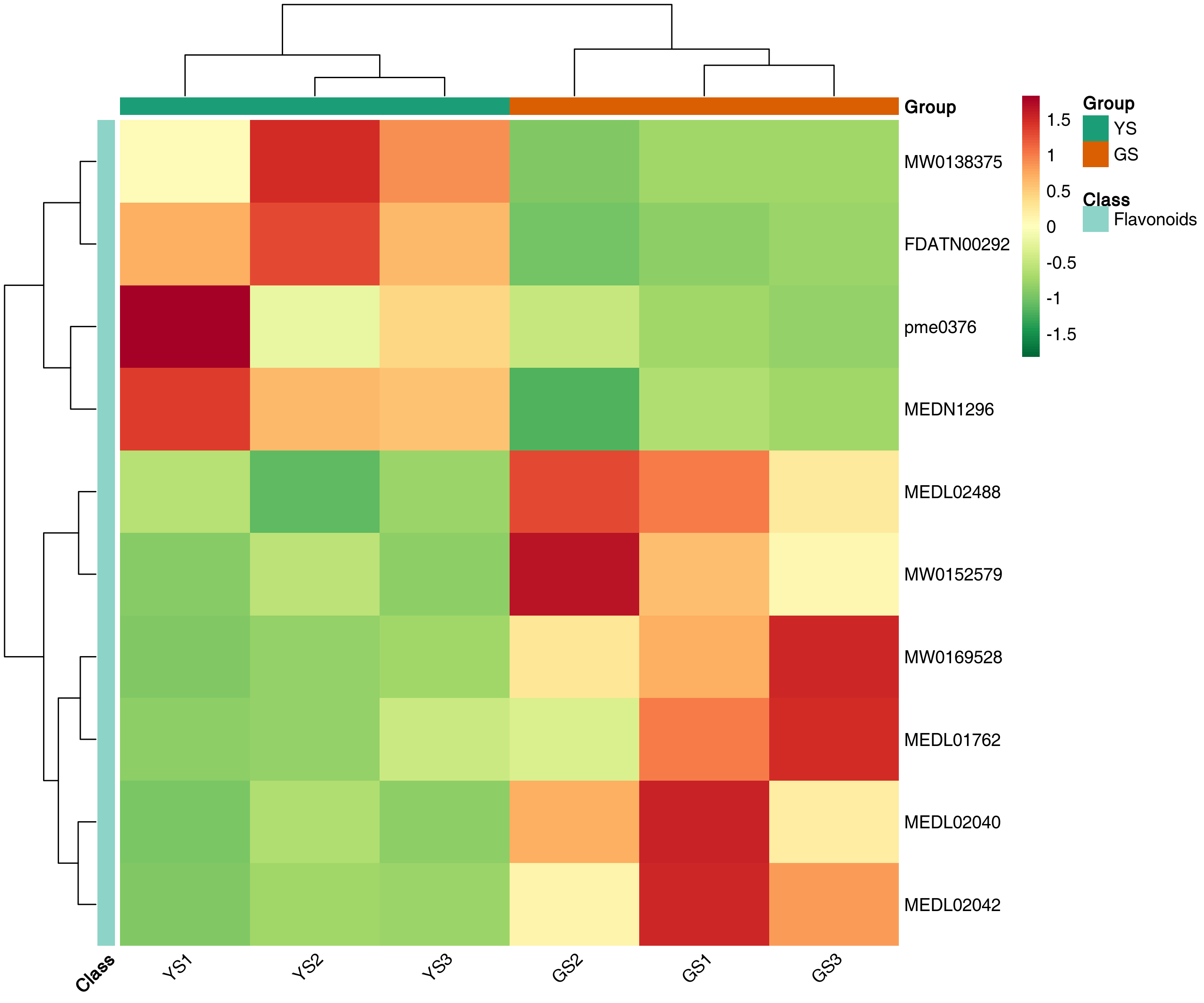

Supplement: Supplementary file 9 [file Image9.png]

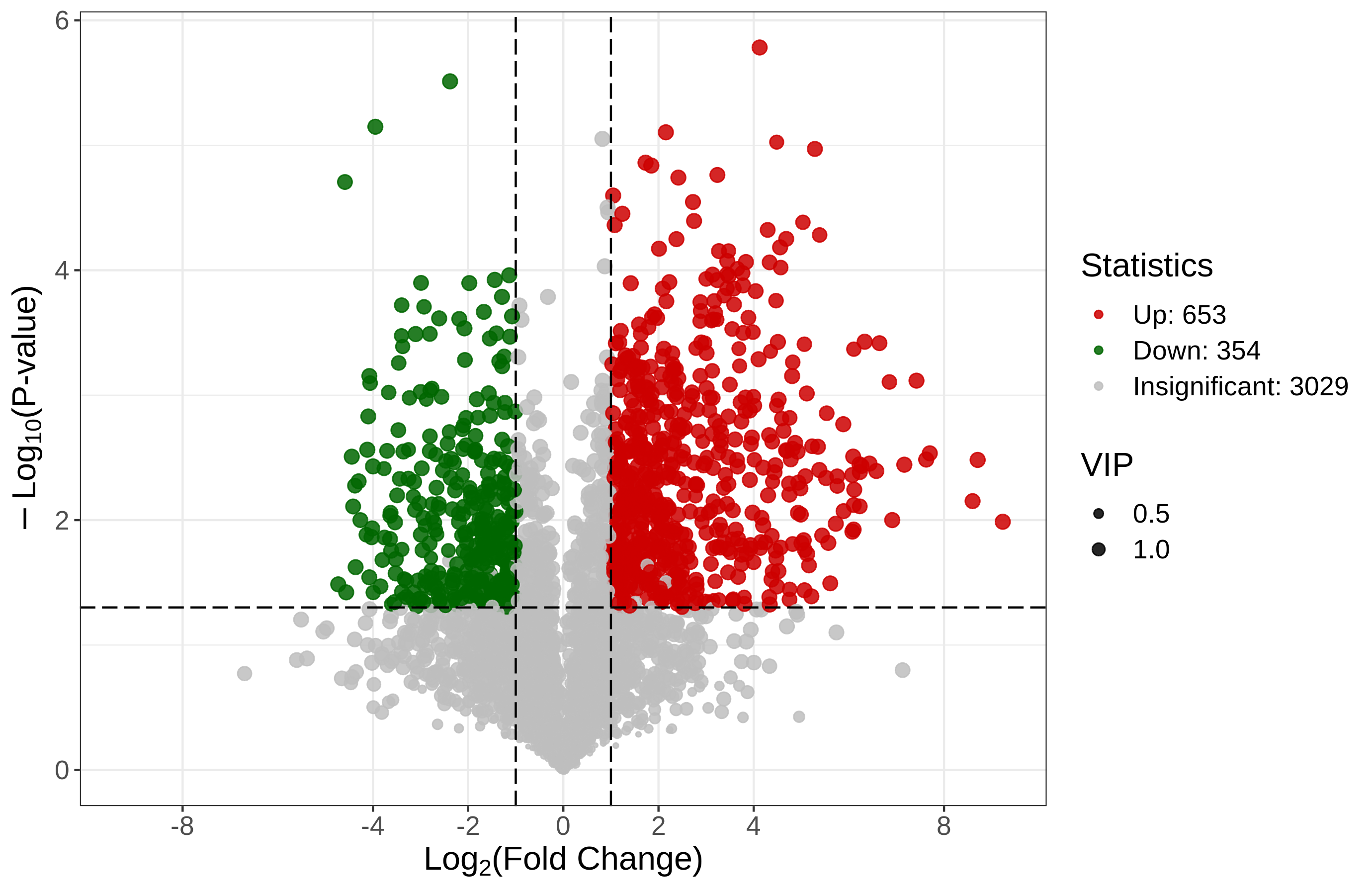

Supplement: Supplementary file 10 [file Image10.png]

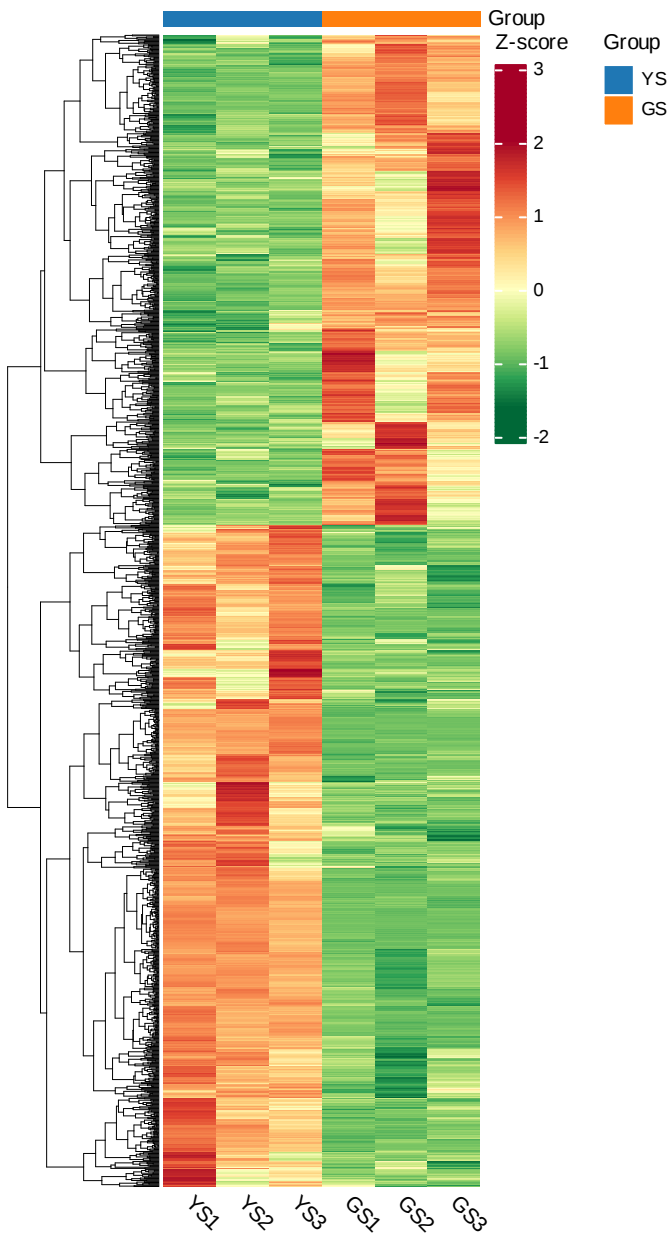

Supplement: Supplementary file 22 [file DataSheet1.pdf]
